# Countering AI-powered disinformation through national regulation: learning from the case of Ukraine

**DOI:** 10.3389/frai.2024.1474034

**Published:** 2025-01-07

**Authors:** Anatolii Marushchak, Stanislav Petrov, Anayit Khoperiya

**Affiliations:** ^1^International Information Academy, Kyiv, Ukraine; ^2^The Institute of Special Communication and Information Protection of the National Technical University of Ukraine "Ihor Sikorsky Kyiv Polytechnic Institute", Kyiv, Ukraine; ^3^Deputy Head of the Center for Countering Disinformation of the National Security and Defense Council of Ukraine, Kyiv, Ukraine

**Keywords:** disinformation, artificial intelligence, law, regulation, prevention, detection, response

## Abstract

Advances in the use of AI have led to the emergence of a greater variety of forms disinformation can take and channels for its proliferation. In this context, the future of legal mechanisms to address AI-powered disinformation remains to be determined. Additional complexity for legislators working in the field arises from the need to harmonize national legal frameworks of democratic states with the need for regulation of potentially dangerous digital content. In this paper, we review and analyze some of the recent discussions concerning the use of legal regulation in addressing AI-powered disinformation and present the national case of Ukraine as an example of developments in the field. We develop the discussion through an analysis of the existing counter-disinformation ecosystems, the EU and US legislation, and the emerging regulations of AI systems. We show how the Ukrainian Law on Counter Disinformation, developed as an emergency response to internationally recognized Russian military aggression and hybrid warfare tactics, underscores the crucial need to align even emergency measures with international law and principles of free speech. Exemplifying the Ukrainian case, we argue that the effective actions necessary for countering AI-powered disinformation are prevention, detection, and implementation of a set of response actions. The latter are identified and listed in this review. The paper argues that there is still a need for scaling legal mechanisms that might enhance top-level challenges in countering AI-powered disinformation.

## 1 Introduction

Over the past decade, digital technology and media have advanced enormously. While these developments benefit individuals and societies, driving economic and social development, they also open the door to information manipulation at scale. Benefitting from the pervasive use of social media (SM) worldwide, perpetrators systematically spread disinformation to destabilize societies, interfere in state governance, and radicalize groups. Addressing these advancements and their impact on states and individuals is a pressing issue for legislators at both national and international levels.

The complexity inherent to countering disinformation campaigns from the legal perspective stems from the juxtaposition between the need to regulate cyber operations and the limits of applying regulations and restrictions on freedom of speech. It is not yet clear what regulations are to be applied to cyber operations (including disinformation), especially under the existing international law frameworks.

On the one hand, such operations could be viewed as a part of espionage activities, which are not directly prohibited by international law (Rosen et al., 2023). On the other hand, they could be assessed as a breach of national sovereignty and be sanctioned under national and international law. There is another element adding to the complexity of the issue – given that democratic states constitutionally guarantee access to information and freedom of speech to their citizens, the extent of law applicability in the outlined scenario is not clear. Unrestricted access to information lies at the core of democratic regimes, as it is believed to enable their citizens to participate freely and fairly in the politics and civic life of the nation. At the same time, some of the existing international law frameworks provide the possibility of developing and applying legislative measures to counteract disinformation. For instance, the General Comment on Article 19 of Civil and Political Rights (ICCPR) provides that “When a State party invokes a legitimate ground for restriction of freedom of expression, it must demonstrate in specific and individualized fashion the precise nature of the threat and the necessity and proportionality of the specific action taken” (Refworld, 2024a).

Given that disinformation campaigns are disseminated predominantly through SM, special attention must be paid to this domain. The threat of disinformation for national security is growing because of perpetrators’ use of artificial intelligence (AI) tools to create and disseminate false and manipulative messages. In particular, fake news websites, AI-generated personalities, and fraudulent accounts are all used to spread harmful narratives. AI-powered social bots can sense, think, and act on SM platforms similar to humans (Hajli et al., 2022).

At the moment, SM regulation is in incipient stages and varies from state to state. It mostly relies on the legal measures developed in the countries of origin (US or China) over the activity of the very large online platforms (VLOPs). According to Stiglitz, a Nobel laureate in economics and a professor at Columbia University “Big Tech’s trade-pact ploy is to create a global digital architecture where America’s digital giants can continue to dominate abroad and are unfettered at home and elsewhere (Stiglitz, 2024). The complexity of VLOPs’ corporate regulation is an additional constraint for national governments to address disinformation on SM. Tackling AI-powered disinformation campaigns requires a multipronged approach involving cyber operations and freedom of speech regulations as well as AI-specific legislation and regulation.

This illustrates further that the constraints to addressing the aftermath of disinformation are that in democratic societies, legislation lags far behind the innovation of emerging technology due to the need for consensus decision-making and the lack of technical expertise possessed by legislators. The mitigation of disinformation and its consequences for national security is dependent on freedom of speech guarantees as well as privacy protection regulations. Still, states must ensure that media and SM are free from malign interference and that civil society participates in public space without disinformation, by enacting mechanisms that distinguish the truth from fiction.

Researchers contributed to defining the most appropriate division of the responsibilities between governments, industry, and civil society while addressing disinformation. Namely, Hamilton (2021) recognizes legal exemptions from fundamental freedom of speech based upon National Security concerns, analyzing the existing practice of content moderation. She defined “the modern free speech triangle” (nation-states, SM companies and users) in the context of responsibility for online content production, amplification, and rule creation and enforcement. Others (Peukert, 2024) pay attention to the justification and the challenges posed by anti-disinformation measures, including the current regulation of counter-disinformation in the EU and the US. Comprehensive analysis of the emerging EU anti-disinformation framework based on the Code of Practice on Disinformation and the Digital Services Act, aimed at minimizing the distribution of false or misleading information has also been conducted (Cavaliere, 2022).

Additionally, scientists identify two strategies for bolstering global AI governance in light of these collaboration issues, which are particularly insightful for our research. These are (a) creating new, centralized international AI institution(s) and (b) enhancing the capacities and coordination of already-existing organizations (Roberts et al., 2024). It has been argued (Roberts et al., 2024), that it is more politically acceptable and practical to fortify the weak “regime complex” of international organizations as they currently stand. Some concerns related to these technologies can be mitigated by inclusive and mutually reinforcing policy change, which in turn would be supported by improved coordination and capabilities amongst the current international organizations controlling AI.

In practical terms, the urgency of the issues introduced above is exemplified at least since Russia’s annexation of Crimea. In the context of Russia’s full-scale invasion of Ukraine, the Ukrainian government and international experts (Tregubov, 2021) recognize that Russian agents spread the most destructive disinformation developed with AI tools, also known as “real-time” deepfakes. The Atlantic Council, a think tank based in Washington DC, underscores that “the quest to find the right balance between free speech and security will shape and define the decades ahead… and Ukraine’s experience should certainly be part of this global conversation.” In this review, our aim is to provide the Ukrainian approach to legal regulations to counter Russian disinformation campaigns, particularly amplified by the use of AI.

This way, the goal of the paper is to address two concerns. The first pertains to the discrepancy between the pace at which AI technology is advancing and the pace at which national government apparatuses can respond, often hampered by the legal provisions inherent in national laws. The second is to discuss the nexus of security concerns and other legal principles, especially in democracies like Ukraine to address AI-powered disinformation.

The paper is divided into three main parts. Section 1 is dedicated to the interconnections between AI and disinformation. Here, both AI usage to spread disinformation and AI-based solutions to address disinformation are discussed. In Section 2 the paper identifies the challenges arising from counter-disinformation and AI regulations in EU, US, and Ukraine with additional emphasis on Foreign Information Manipulation and Interference (FIMI). Section 3 maps the law’s applicability in AI and counter disinformation nexus. The self-regulation or binding legal regulations approaches for VLOPs are examined. Section 3 also proposes a set of preventative, detection, and response actions to address AI-powered disinformation.

## 2 AI and disinformation: interconnections

### 2.1 How AI is used to spread disinformation

As modern reality shows, AI can be both a tool for objective information reaching the masses and a powerful tool for spreading false or manipulative messages. This opens up wide opportunities for those who intend to manipulate public opinion, control the information space, and influence political processes. People can become victims of disinformation without the ability to distinguish truth from manipulation. This is especially dangerous for political and public discourse, where the accuracy of the information can determine the future of a country and its citizens. For the Ukrainian government tackling Russian disinformation sometimes even means saving the lives of the country’s citizens.

AI tools are actively used to exert destructive information influence. In particular, fake news websites or voiced AI-generated virtual personalities are used to spread manipulative information. Fake accounts of non-existent people are also being created to promote information needed by the perpetrator. AI can provide more comprehensible and accurate information than people, but it can also generate more persuasive disinformation (Spitale et al., 2023).

In recent years, deepfakes have often been used to exert destructive influence. Ukrainian government notes that Russian propaganda spreads the most dangerous type of deepfakes, called “real-time” deepfakes. “Real-time” deepfake technology poses a serious threat to the information sphere due to its ability to create fake videos instantaneously, potentially deceiving both the public and political elites of various countries, thereby influencing decision-making processes. This technology allows for quick and almost undetectable changes to content, including the faces of politicians or other influential figures, manipulating words and images. In light of such deep-fake capabilities, the threat of trust in information becomes critical for society.

One example of effective use of “real-time” deepfake technology is the propaganda show “Show ViL” by Russian pranksters “Vovan” (Vladimir Kuznetsov) and “Lexus” (Aleksei Stolyarov), known for their conversations with high-ranking officials from various countries. One of their notable uses of “real-time” deepfake was in a conversation with Krišjānis Kariņš, the former Prime Minister of Latvia (Rutube, 2024a) or former President of Poland Aleksander Kwaśniewski (Rutube, 2024b), where they discussed controversial and sensitive political and geopolitical issues. They used “real-time” deepfake technology to make video calls, pretending to be political figures from certain African countries.

The pranksters conduct video calls with the targeted persons on behalf of other public figures whose images are generated online by AI. Moreover, the generated images and sound are of such high quality that they do not raise any doubts among the victims of the prank. The content obtained in this way is published by the Russian side in the public domain to discredit the persons who became the target of such a propaganda “prank.”

Ukrainian Center for Countering Disinformation (CCD), the working body of the National Security and Defense Council of Ukraine (NSDC) uncovered a damaging information campaign against President Volodymyr Zelenskyy constructed with the help of AI. The report “Information Influence Campaign in the African Information Space” (Center for Countering Disinformation, 2024) provides details of the campaign and is a striking illustration of foreign information manipulation and interference (FIMI). Using resources from African media, the campaign’s primary objective was to denigrate Zelenskyy and Ukraine in the eyes of international allies. The quasi-state actors disseminated several bogus films and articles under false names through certain African media sources with anti-Ukrainian rhetoric.

A prominent case of this campaign is about the alleged ownership of a villa in Egypt by President Zelenskyy’s family. On August 22, 2023, the Nigerian media outlet Punch published an article titled “A Luxury Villa Owned by President Zelenskyy’s Family Found on the Coast of Egypt.” The material, citing an investigation by “Egyptian journalist” Mohammed Al-Alawi from August 20, 2023, mentioned a villa in the Egyptian resort town of El Gouna, allegedly belonging to Zelenskyy’s mother-in-law, Olga Kiyashko. However, the CCD found that the Zelenskyy family does not own any property in Egypt. Moreover, no evidence was found to confirm the existence of Mohammed Al-Alawi, indicating a high probability of AI tools being used to create this persona. Additionally, Mohammed Al-Alawi’s YouTube channel (Center for Countering Disinformation, 2024) creation and the video about the villa were dated the same day. This also indicates manipulative tactics.

The dangers of AI technologies in spreading disinformation should be considered broadly, factoring in not only the creation and manipulation of content but also the use of AI to disseminate it, amplify the likelihood of preexisting threats, and profile users with greater precision. AI systems are currently being abused in several fields, and if they are employed more widely, there will be greater opportunities for abuse. On such misuses, decision-makers will feel obliged to step in, but it can be challenging to select the best set of responses (Anderljung and Hazell, 2023). Developers would have a strong incentive to set up organizational procedures for guaranteeing honest and efficient reporting if regulations imposed legal penalties for careless or intentional misreporting. Regulators-approved independent auditors may also be able to help find instances of misreporting (Kolt et al., 2024).

However, it might still be challenging to match the fundamental rights criteria with the decision models of more sophisticated algorithms (Buiten, 2019). Policymakers ought to concentrate on the dangers that they wish to lower. It is demonstrated that defining the primary sources of relevant risks – specific technological strategies (like reinforcement learning), applications (like facial recognition), and capabilities (like the capacity to engage physically with the environment) – better satisfies the requirements for legal definitions (Schuett, 2023).

The legal system faces both conceptual and practical issues as a result of the distinctive qualities of AI and how it can be developed (Scherer, 2015). According to Viljanen and Parviainen (2022), the five layers of AI law are the following: data rules that govern data use, application-specific rules that target AI applications or application domains, general AI rules that apply to a broad range of AI applications, application-specific non-AI rules that apply to specific activities but not to AI specifically, and general non-AI rules that apply generally and across domains. The last two layers are counter-disinformation legislation in our case.

Worries about AI safety have arisen because of AI systems’ unpredictability, explainability, and uncontrollability. Because of the complexity of AI systems, limitations in human understanding, and elusiveness of emergent behaviors, it is impossible to predict certain capabilities with any degree of accuracy (Yampolskiy, 2024). Moreover, gaining an awareness of various rule complexes, their dynamics, and regulatory modalities is necessary to comprehend the regulatory environment around AI (Viljanen and Parviainen, 2022). Governments must recognize the significance of adopting a regulatory framework that optimizes AI’s advantages while accounting for its hazards. This might entail classifying and categorizing risks according to relevant legal frameworks and country situations, when applicable (AI Safety Summit, 2023).

Some scientists find compute governance to be a significant approach to AI governance. Tens of thousands of sophisticated AI chips are needed to train sophisticated AI systems; these chips cannot be purchased or used covertly. AI chips can be supplied to or taken away from specific actors and in certain situations due to their physical nature. Moreover, it is measurable: it is possible to measure chips, their attributes, and their utilization. The extremely concentrated structure of the AI supply chain further enhances the compute’s detectability and excludability (Heim et al., 2024). Finally, if mitigations are not implemented for AI-based disinformation including legal regulations, interactive and compositional deepfakes have the potential to bring us closer to a post-epistemic world in which it will be impossible to tell fact from fiction (Horvitz, 2022).

### 2.2 AI-based solutions to address disinformation

The issue of countering AI-powered disinformation is extremely important, but it is greatly complicated because of the rapid development of the technology for generating deepfakes, including mentioned “real-time” deepfakes. Today, we know for certain about digital visual evidence that may indicate interference with AI content: occlusions, imperfect edges of visual masks, color and light mismatch, etc. However, it is predicted that these image defects will be eliminated in the near future, as AI technologies are developing extremely dynamically. A wide range of significant and urgent threats associated with AI are being discussed more and more by AI specialists, journalists, policymakers, and the public (Center for AI Safety, 2024).

There are already technical, legal, regulatory, and educational approaches to counter disinformation in Ukraine – some of which have already been implemented and some of which are just emerging – that can help reduce the level of threat associated with the use of AI. Looking at the case of Ukraine, it is worth paying attention to the activities of some of Ukrainian AI-based platforms (Osavul, 2024) which effectively help to detect destructive information influence campaigns in their early stages. Their capabilities are powered by CommSecure and CIB Guard software. CommSecure enables to detect specific narratives in messages on social networks and communities, such as public groups in messengers. This ensures that potentially dangerous information flows are quickly identified and analyzed. CIB Guard, on the other hand, specializes in analyzing public user pages, identifying bots, and determining whether they act in a coordinated manner. This approach allows to quickly recognize coordinated campaigns that may be aimed at manipulating public opinion or spreading disinformation.

## 3 Counter disinformation and AI regulations

First of all, we would like to mention that the current international law principle of sovereignty clashes with the cross-border nature of cyberspace and disinformation operations. The UN Open-Ended Working Groups (OEWG) as the multi-lateral forum for cyber diplomacy has not yet provided the recommendations applicable for countering the AI-powered disinformation.

Historically, one of the first cases of legally defined misconduct in disinformation is dated January 2019. Then the US a company that created fake SM profiles to make millions of dollars in revenue settled a case with the New York state attorney. The settlement is the first case in which law enforcement has concluded that selling fake SM activity is illegal (Funke and Flamini, 2024).

As stated above, VLOPs and their platforms are usually to some extent dependent on the laws of the host countries. However, their terms of service are devised based on the legal system of the country of origin, currently predominantly the US and China. VLOPs’ intention to develop internal counter-disinformation mechanisms including AI-powered solutions can be affected by commercial and geopolitical apprehensions, e.g., risks of retributory regulation or losing access to market in the host country.

Contrary to the approach to making VLOPs responsible for addressing the disinformation Hamilton’s (2021) opinion is that “as a strictly legal matter, there is no reason for the platforms to have developed the elaborate content moderation systems they currently run.” VLOPs faced the risks of “wasting” time, finance, and human resources on addressing disinformation by monitoring their networks, detecting fake news and even losing their users if the moderation gives rise to public debate. Thus, VLOPs used to be reluctant to identify perpetrators of disinformation. Nevertheless, VLOPs under pressure or in collaboration with governments, predominantly the US and China, started to develop detection and suspension initiatives, including those relying on AI, aimed at bots and botnets, as well as users exposed to disinformation, reinforcing the visibility of reliable content produced by trustworthy media and fact-checking sources, and vice versa reducing visibility (Santa Clara University, 2024) or suspension of sites’ disinformation content.

At this point various legal approaches to counter disinformation have been put in place, helping reduce the level of threat associated with the use of AI. According to many reports, legislation governing AI is still in its infancy, with few statutes and other regulatory tools governing the creation and application of AI (Viljanen and Parviainen, 2022). The traditional conundrum of defining AI is exacerbated by the fact that our knowledge of natural intelligence is still incomplete (Mahler, 2021). Overcoming the present shortcomings in global AI governance is complicated by first-order cooperation issues resulting from interstate competition and second-order cooperation issues arising from dysfunctional international institutions (Roberts et al., 2024).

Recently several declarations have been produced at the international level. For instance, UK Bletchley Park hosted the first global summit on frontier AI safety (Artificial intelligence, 2023). NATO Washington Summit Declaration adopted on 10 July 2024 also mentioned the intention of the NATO member-states to develop individual and collective capacity to analyze and counter hostile disinformation operations (NATO, 2024). Additionally, in May 2024 at the AI Safety Summit in Seoul the following AI businesses pledged to uphold a set of international guidelines for AI safety known as the Frontier AI Safety Commitments: Amazon, Anthropic, Cohere, Google, IBM, Inflection AI, Meta, Microsoft, Mistral AI, Open AI, Samsung, − Technology Innovation Institute, xAi, Zhipu.ai (Zhipu.ai is a Chinese company backed by Alibaba, Ant and Tencent) (AI Seoul summit, 2024).

Finally, authorities like Ukrainian CCD to counteract disinformation have also been developed along regional lines. They’ve also established the basis of coalitions with such entities as the EU Intelligence and Situation Centre (INTCEN), EU East StratCom Task Force with the flagship project EUvsDisinfo, the Helsinki European Centre of Excellence for Countering Hybrid Threats (counter disinformation capacity), and the NATO Strategic Communication Excellence Centre, among other.

### 3.1 The EU regulations

Holding EU candidate status Ukraine is actively harmonizing its legislation with EU legislation and regulations, particularly in measures to counter AI-powered disinformation. The need to legally address the threat is based upon recent EU legal decisions. For instance, advanced disinformation/influence operations campaigns coupled with abuse of AI are listed as a revised line-up of the emerging cybersecurity threats to have an impact by 2030 in the EU (ENISA, 2024). Additionally, the European Union Council on May 21, 2024, approved two documents pertaining to disinformation management – the Future of EU Digital Policy (Council of the European Union, 2024a) and Council conclusions on democratic resilience: safeguarding electoral processes from foreign interference (Council of the European Union, 2024b). The first document seeks to establish the framework for the next 5 years of digital policymaking and disinformation is listed as one of the detrimental or illegal occurrences that must be combated while promoting entrepreneurship, innovation, and the growth of the capital market. The durability of democracy and preventing outside intervention in electoral processes are the main topics of the second document (Council of the European Union, 2024b). It also provides a comprehensive overview of the legislative, non-legislative, and institutional tools that the EU has established. Both these documents stress the importance of further legal developments in counter disinformation realm. Consequently, they will be reflected to some extent in Ukrainian regulation.

#### 3.1.1 European media freedom act

Some norms of the European Media Freedom Act (EMFA) (European Media Freedom Act, 2024) will be harmonized with the Ukrainian legislation as well. As Gamito (2023) stated before enacting EMFA the “online media freedom” was regulated domestically due to the threats of disinformation, without having in mind the European internal market. From the Ukrainian perspective, particularly interesting will be powering the European Board for Media Services (the Board) to engage in dialogue with VLOP to monitor adherence to self-regulatory initiatives aiming to protect users from harmful content, including FIMI. The EMFA emphasizes “insufficient tools for regulatory cooperation between national regulatory authorities or bodies.”

With reference to the EU Code of Practice on Disinformation (European Commission, 2024) the EMFA obliges the Board to organize a structured dialogue between providers of VLOPs, representatives of media service providers, and representatives of civil society. This is to foster access to diverse offerings of independent media including as regards the moderation processes by VLOPs and monitor adherence to self-regulatory initiatives to protect users from harmful content, including disinformation and FIMI (Gamito, 2023). The implementation of these norms into Ukrainian legislation is of vital importance to counter Russian AI-powered disinformation at VLOPs platforms.

#### 3.1.2 European AI act

Another major document is European AI Act (European Parliament, 2024) that contains several provisions on disinformation that need to be reflected in Ukrainian legislation. The European AI Act^1^ directly addresses systemic risks posed by general-purpose AI models. These risks include the risks from the facilitation of disinformation. Legally significant is the AI Act definition of “deepfake”^2^ as AI-generated or manipulated image, audio, or video content that resembles existing persons, objects, places or other entities or events and would falsely appear to a person to be authentic or truthful (European Parliament, 2024).

Moreover, the European AI Act^3^ stipulates the obligations placed on providers and deployers of certain AI systems to enable the detection and disclosure that the outputs of those systems are artificially generated in particular as regards the obligations of providers of VLOPs or very large online search engines to identify and mitigate the dissemination of content that has been artificially generated or manipulated, including through disinformation (European Parliament, 2024). Additionally, the AI Act^4^ stipulates that deployers, who use an AI system to generate “deepfakes,” should also clearly and distinguishably disclose that the content has been artificially created or manipulated by Labeling the AI output. The compliance with this transparency obligation should not be interpreted as indicating that the use of the system or its output impedes the right to freedom of expression and the right to freedom of the arts and sciences (European Parliament, 2024). The requirement to label content (p. 136 of the AI Act) generated by AI systems is without prejudice to the obligation in Article 16 (Peukert, 2024) of Regulation (EU) 2022/2065 (European Union law, 2024) - providers of hosting services shall process any notices that they receive under the mechanisms to allow any individual or entity to notify them of the presence on their service of specific items of information that the individual or entity considers to be illegal content.

These norms will not be binding for Ukraine and VLOPs’ activities in the country until Ukraine becomes an EU member. Thus, the norms should be reflected in Ukrainian regulations on digital services and AI.

Another example of non-legislative regulation in the EU is the Code of Practice on Disinformation of the EU. The Code of Practice was initially signed by Facebook, Google as well as Twitter, Mozilla, advertisers, and parts of the advertising industry, Microsoft and TikTok (European Commission, 2024). In the Code, the signatories recognized “the fundamental right to freedom of expression and to an open Internet, and the delicate balance which any efforts to limit the spread and impact of otherwise lawful content must strike” (European Commission, 2024). Special attention in the Code is given to the case law of The Court of Justice of the European Union (CJEU) on the proportionality of measures designed to limit access to and circulation of harmful content (European Commission, 2024).

It is worth mentioning that Russia has been expelled from the European Court of Human Rights (ECHR) so is no longer a state party to rulings on HR violations by that court, including on Article 10. However, in the recent ECHR Judgment dated 22 October 2024 in the case of Kobaliya and others v. Russia the Court held that there had been violations of the right to freedom of expression, the legislative framework had become considerably more restrictive since 2012, and had moved even further from Convention (European Convention on Human Right) standards” (European Court of Human Rights, 2024). As a result, such judgments of ECHR will not be currently legally executed in Russia. These make the ECHR mechanism not feasible for counter-disinformation efforts.

Contrary, the CJEU judgments could be a more efficient tool to counter Russian disinformation campaigns based upon the EU’s regime imposing restrictive measures on Russian individuals and entities due to Russia’s aggression against Ukraine (Pingen and Wahl, 2024).

Finally, the Code of Practice principles lay out two parts to consider when developing a legal framework for counteracting AI-powered disinformation: (1) no authority for governments to compel content moderation; (2) the content moderation could not be executed only on the basis that messages are thought to be “false.”

### 3.2 Combating FIMI and counter AI-powered disinformation

AI development and accessibility have generated a lot of discussion, especially when it comes to how they could be abused for malevolent objectives in disinformation and FIMI. Actors in the FIMI rapidly started experimenting with these new tools to produce simulated media (EEAS, 2024a). The FIMI concept was developed to intercept various tactics used to manipulate society and protect the information space (EEAS, 2024b). Substantial disinformation efforts aiming at undermining EU members were uncovered following the start of Russian aggression in Ukraine in 2014, marking the first substantial moves in the FIMI approach. Officially implemented as part of EU policy, the specific FIMI effort was part of the European Action Plan Against Disinformation (European Union, 2018), which was enacted in December 2018. This action plan called for the establishment of a quick alert system to facilitate coordination amongst EU member states and the development of an operational task force, known as the East StratCom Task Force, to battle disinformation.

FIMI is defined by the European External Action Service (EEAS) as a pattern of behavior that jeopardizes or may have a detrimental effect on political structures, procedures, and ideals. These kinds of actions are planned, deliberate, and manipulative. While the main objectives of the EU are to safeguard the information space from harmful external influences, ensure transparency and honesty in information exchange, and strengthen democratic institutions by enhancing resilience to disinformation, the primary goal of this behavior is to influence public opinion, undermine democratic processes, and destabilize society (FIMI-ISAC, 2024).

Through collaboration with NGOs, academic institutions, and media from Africa and Latin America, the European Union and the United States are strengthening their preparedness for FIMI. To establish a multilateral community with the goal of enhancing collaboration in response to FIMI, they host workshops. To gain a deeper understanding of the disinformation strategies and narratives that are used in these areas, as well as the capacity of local stakeholders to counter them, they also gather data from fact-checking networks. Training in digital competency, media literacy, and development funding channels all improve support for capacity-building. It is also highlighted that maintaining a free and diverse media environment is crucial for countering disinformation and other FIMI (EEAS, 2024c).

Combating FIMI is essential to preserving national security, upholding democratic processes, and guaranteeing social cohesion within the EU, US, and other countries including Ukraine.

### 3.3 US legislation and regulation

As mentioned above the VLOPs’ terms of service are developed based upon the legal system of the country of origin, mostly the US one. It is worth paying attention to the US regulations in order to figure out the principles used by VLOPs while addressing AI-powered disinformation.

The US legislation on AI-powered disinformation, which is important for the Ukrainian government to address Russian disinformation domestically and abroad, was drafted in the form of The Deepfakes Accountability Act introduced in June 2019 to combat the spread of disinformation through restrictions on deep-fake video alteration technology (Clarke, 2019). Additionally, the United States has introduced legal regulation with the Algorithmic Accountability Act (Wyden, 2022).

On the other hand, proposals for legislative ways to address disinformation in SM networks that give rise to national security concerns could contribute to the ongoing debate in the US and worldwide on the matter of how and by what authority SM networks could be regulated. Taking into account the evolving moderation of online expression, the gap in legal and regulatory terms regarding VLOPs’ responsibility affects the national interests of other democratic states. The complexity of the problem derives also from the evolving opportunities for disinformation spurred by technology – for instance, mentioned above “deep-fakes” produced with the use of AI.

However, the US Constitution and case law have not been particularly consistent in the application and interpretation of freedom of speech restrictions. The US Constitution, First Amendment only applies to laws enacted by Congress and to local, state, or federal government agencies, but not to the actions of private VLOPs. Thus, the responsibility of VLOPs regarding freedom of speech and counter disinformation dissemination activities are defined predominantly by corporate policies. The US and other democracies’ legal approaches to VLOPs were thus far based upon self-regulation. However, new regulations are currently evolving in AI and counter disinformation domain in the US and EU.

The US Deepfakes Accountability Act 2023 established the “Deepfakes Task Force” particularly to advance efforts of the US Government to combat the national security implications of deepfakes (Clarke, 2019).

A historic US Executive Order on Safe, Secure, and Trustworthy AI was also issued in late 2023, requiring systems that are far more sophisticated than those in use today to submit reports (The White House, 2023). Additionally, in the US NIST developed the Artificial Intelligence Risk Management Framework: Generative Artificial Intelligence Profile with the preventative action to identify potential content provenance risks and harms in GAI, such as disinformation, deepfakes (NIST, 2024).

However, the US legislation on counter disinformation is developing the freedom of speech and freedom of VLOPs’ entrepreneurship principles without proper consideration of the national security concerns. For instance, the Disinformation Governance Board Prohibition Act 2023 terminated the Disinformation Governance Board of the Department of Homeland Security (Bice, 2023). Additionally, the Free Speech Protection Act 2023 prohibits federal employees and contractors from directing online platforms to censor any speech that is protected by the First Amendment to the Constitution of the US (Paul, 2023).

As a result of a lack of bilateral US-Ukraine governmental collaboration tools, the Ukrainian government needs to develop legal and operational mechanisms to directly communicate with the VLOPs, based in the US, to counter Russian AI-powered disinformation. In this context, the principles of AI self-regulation, voluntarily committed and introduced in 2023 by several AI developers in the US (The white House, 2024), need to be particularly considered.

### 3.4 Ukraine’s regulation on countering disinformation and AI

To understand the legal prerequisites for countering AI-powered disinformation in Ukraine there is a need to briefly analyze the country’s relevant legislation.

Article 43 of the Ukrainian Constitution recognizes article 19 of ICCPR providing: “the exercise of… rights (to freedom of thought and speech) may be restricted by law in the interests of national security…” (Refworld, 2024b) The Ukrainian Law on Information defines “completeness and accuracy of information” (Bulletin of the Verkhovna Rada, 1992) as one of the basic principles of informational relationships. But there is no answer on the criteria of such information, and consequently no clear legal background for a definition of disinformation. Ukraine needs to establish this definition of disinformation in its national legislation. This is even more important considering the harm to the Ukrainian nationals caused by Russian disinformation campaigns. Executing the right to defense from Russian armed aggression Ukraine has additional justification on the basis of national security to counteract the adversary’s disinformation operations. These included, for example, the May 2017 banning of several Russian websites such as VKontakte, Odnoklassniki, Yandex, and Mail.ru.

The first legal concern for Ukraine was to establish and enhance governmental authorities in charge of addressing disinformation. The necessity for the Ukrainian government to guard against foreign meddling in SM is obvious, but it could still potentially impinge upon freedom of expression and result in direct censorship. Another action the Ukrainian government made to counteract Russian propaganda and disinformation was the creation of the appropriate governmental bodies (e.g., Ministry of Culture and Strategic Communications). In March 2021, the Center for Strategic Communications and Information Security was established with the main objective of joint development of mechanisms to counter disinformation, together with international partners. Further, in line with the National Security Strategy of Ukraine, the CCD was established. The primary goal of this step was to ensure effective countering of propaganda and destructive disinformation campaigns. In May 2021 the President of Ukraine signed the regulation establishing the CCD (President of Ukraine, 2024a). The framework of this document establishes that the CCD is tasked with identifying and countering disinformation, propaganda, and destructive informational influence efforts and campaigns, as well as preventing attempts to manipulate public opinion (President of Ukraine, 2024b).

With regard to the existing Ukrainian legislative experience in the field of countering disinformation, Ukraine is gradually moving toward developing its own approach to countering the ever-growing information threats. Thus, in recent years, the Ukrainian legal framework has been supplemented by the Laws of Ukraine “On Cloud Services” and “On Stimulating the Development of the Digital Economy in Ukraine,” as well as the Law “On Media.”

At the same time, it should be emphasized that Ukraine has legislation in place to counter disinformation that provides guarantees of judicial protection and civil rights, among other things: For instance, Article 32 of the Constitution of Ukraine states: “Everyone is guaranteed judicial protection of the right to refute false information about himself or herself and members of his or her family and the right to demand the withdrawal of any information, as well as the right to compensation for material and moral damage caused by the collection, storage, use and dissemination of such false information” (Refworld, 2024b). Further, the Law on Information, Article 278 of the Civil Code of Ukraine, attempts to balance freedom of speech with the protection of legitimate interests, rights, and freedoms of individuals and legal entities. The Criminal Code of Ukraine (CCU) (Legislation of Ukraine, 2011) provides for criminal liability for certain offenses related to destructive information influence, in particular: Article 259 of the CCU (“Knowingly False Reporting of a Threat to the Safety of Citizens, Destruction or Damage to Property”) provides for liability for knowingly false reporting of preparations for an explosion, arson, or other actions threatening the death of people or other severe consequences; Article 436 of the CCU (“Propaganda of War”) provides for liability for public calls for aggressive war or for initiating a military conflict, as well as for the production of materials with calls to commit such actions for the purpose of their dissemination or distributing such materials. However, none of these legal acts provide for the liability of VLOPs and search services for the dissemination of disinformation, particularly with the use of AI.

In August 2023, the Parliament of Ukraine Verkhovna Rada adopted the “European Integration” law “On Digital Content and Digital Services,” which introduced the terms “digital content” and “digital service.” This law is aimed at protecting consumer rights when purchasing and using digital content or services. However, this law does not include a human rights part, as the European Digital Services Act (DSA) does. This opens up opportunities for Ukraine to define the legal relationship between VLOPs and users in the context of countering AI-powered disinformation. It should also be emphasized that as part of the implementation of measures to synchronize and harmonize Ukrainian legislation with that of the EU, the Ministry of Digital Transformation and the Verkhovna Rada Committee on Humanitarian and Information Policy are taking active measures to implement the DSA, in particular by amending the Law of Ukraine “On Media.”

Specifically, the DSA envisages new responsibilities for platforms and the empowerment of users, which, among other things, will include the following measures for:

- Countering illegal content, goods and services: Online platforms should provide users with the ability to flag illegal content, including goods and services. Moreover, platforms should cooperate with “trusted flaggers,” specialized organizations whose alerts should be prioritized by the platforms.
- Protecting minors: including a complete ban on targeting minors with ads based on profiling or their personal data.
- Providing users with access to a complaint mechanism to appeal decisions on content moderation.
- Publishing a report on content moderation procedures at least once a year.
- Providing users with clear terms and conditions, including the basic parameters on which their content recommendation systems operate.
- Appointing a contact person for government authorities and users.

The implementation of the DSA in Ukraine will bring significant benefits in line with the countermeasures against AI-powered disinformation. First, it will increase the transparency of online platforms in Ukraine by forcing them to be open about their content moderation algorithms and procedures, providing users with information about the reasons for content removal or account blocking, and publishing annual reports. Second, it will strengthen the protection of users’ rights by providing them with the opportunity to appeal content moderation decisions through special complaint mechanisms, which will help protect the rights to freedom of speech and personal information. Third, the law will help fight illegal content by allowing users to flag illegal content and cooperate with “trusted flaggers.” The Concept on development of AI in Ukraine was approved by order of the Cabinet of Ministers of Ukraine on December 2, 2020 No. 1556 (The Parliament of Ukraine, 2020), however, there is no law on AI yet in Ukraine.

Finally, it is worth mentioning that Ukraine is actively working to put the FIMI standard into practice right now. The European External Action Service EEAS trained the CCD of the National Security and Defense Council of Ukraine in October 2023, working with the EU Advisory Mission in Ukraine (EUAM Ukraine) to introduce them to the FIMI approach and evaluate the suitability and effects of implementing FIMI for the CCD. The CCD receives from EEAS a complimentary dedicated instance of Open Cyber Threat Intelligence (OpenCTI), a knowledge management and sharing platform for FIMI and cyberspace.

Summing up, AI-powered disinformation campaigns undermine democratic processes, but is it enough to apply the freedom of speech exemptions based on national security concerns? Additionally, what could and should be the legal mechanism to clearly define national interests on a case-by-case basis? The answers are not obvious because of the nexus of domestic and international issues involved and the differences within legal systems. In the current circumstances, the Ukrainian government had legitimate grounds to proceed with banning AI-powered disinformation on VLOPs’ platforms based on national security concerns. It is already recognized worldwide that Russia violated international law, and Ukraine had a right to impose anti-disinformation measures as a proportionate self-defense in line with international and domestic law.

## 4 Discussion: law applicability in AI and counter disinformation nexus to address national security concerns

### 4.1 Self-regulation or binding legal regulations for VLOPs

There is a complex legal issue centering on the responsibility of VLOPs, with regard to freedom of speech and the laws applicable to certain disinformation dissemination activities. As described above, Ukraine is dependent on corporate policies defining the responsibility of the US-based VLOPs regarding freedom of speech and counter disinformation, including AI-powered.

Despite professing commitment to free speech, the main objective of these companies is profit. The more customer attention VLOPs attract, the more advertising revenue is gained. Provided that disinformation is not defined as illegal and tends to spread further and faster than verified information, VLOPs can be potentially incentivized to engage in its dissemination. VLOPs faced the risks of “wasting” time, finance, and human resources on addressing AI-powered disinformation by monitoring their networks, detecting fake news and even losing their users if the moderation gave rise to public debate; thus, VLOPs used to be reluctant to identify perpetrators of disinformation.

Interestingly, according to the US Communications and Technology Subcommittee and the Consumer Protection and Commerce Subcommittee (House Committee on Energy and Commerce, 2024), the industry self-regulation has failed. This opinion is seconded by scientists, who believe that self-governance is not able to consistently endure the pressure of financial incentives. Assuming that these incentives will always be in line with the public interest is insufficient given AI’s huge potential for both positive and negative effects. Governments need to start creating efficient regulatory frameworks right away if they want the development of AI to benefit everyone (Toner and Mccauley, 2024). Let us have a look at some VLOPs’ internal policies and trends to counter AI-powered disinformation. VLOPs under pressure or in collaboration with governments, predominantly the US one, started to develop detection and suspension initiatives, including those relying on artificial intelligence, aimed at bots and botnets, as well users exposed to mis- and disinformation, reinforcing the visibility of reliable content produced by trustworthy media and fact-checking sources, and vice versa reducing visibility (Santa Clara University, 2024) or suspension of sites’ disinformation content.

The creation of the Facebook (Meta) Oversight Board was, for instance, a positive step toward setting principles and rules for the VLOP content moderation. However, with no binding law regarding counter-disinformation, the Oversight Board can only solve its flagged concerns based on the Code of Conduct, which does not provide a clause on disinformation, particularly on AI-powered content. Facebook’s (Meta’s) policy criteria of importance to public discourse and the number of individuals impacted is vital from the Ukrainian counter-disinformation perspective, such as in the case of the Kremlin-linked TV channels ban, where the need to prohibit broadcasting on SM platforms presented (Dickinson, 2021). Google legal policies (for instance, YouTube’s) (Google, 2024) stipulate the following legal issues to file a complaint: trademark, counterfeit, defamation, stored music policy, other legal issues and complaints (YouTube, 2024). There is no exact and clear way to counter AI-powered disinformation using legal mechanisms. Even TikTok announces its initiatives to improve platform transparency and prevent covert influence campaigns. The platform claims to have discovered and destroyed networks involved in coordinated acts of inauthentic behavior (Tiktok, 2024).

However, we should agree with Rebecca Hamilton’s opinion (Hamilton, 2021) that, “as a strictly legal matter, there is no reason for the platforms to have developed the elaborate content-moderation systems they currently run.” Another complicated legal issue is that AI developers have motivations that are not in line with the interests of the general population. Developers will probably be pushed by financial incentives to underinvest in safety, which would be especially worrying if frontier AI systems result in significant negative externalities. This motivation mismatch indicates that there is also a need for strict supervision of AI developers. Thus, the only consistent solution at the national and/or international level would be to enact enforcement regulations covering VLOPs’ operations in addressing AI-powered disinformation.

The case of Ukraine shows that rapid action at times has to be taken, and we want to show some examples of how this may be possible on the legal level. Of course, such rapid action is possible in the unprecedented circumstances of limited freedom of martial law. The legal concern is the extent to which governmental authority respects freedom of speech, privacy, and rule of law principles while addressing AI-powered disinformation. National governments should not be the only ones in charge of addressing AI-powered disinformation. Corporations should not be in charge of self-regulation either Marsden et al. (2020) propose co-regulation when businesses create their own user regulations, either separately or together, which must then be authorized by democratically legitimate state legislatures or regulators, who also keep an eye on how well they work. Such an approach could be effective in the Ukrainian realm of law while defending from Russian aggression. Accepting the principle that regulatory policies may be more reversible in AI than in other environments (Carpenter, 2024), we propose a “functional approach” (see Table 1), based upon the analysis of actions required for countering AI-powered disinformation: prevention, detection, and response to such campaigns.

**Table 1:** Responsibility of stakeholders in counter AI-powered disinformation activities.

Actions\Stakeholders	State	VLOPs	Civil society organizations/traditional media/academia	Citizen(s)
Prevention				
Development of reliable news network	Support (S) 3	S2 (*number correlates with the level of involvement from 1 – highest to 3 lowest*)	L (*Leading stakeholder*)	S1
Raising awareness	L	S2	S1	S3
Providing mechanism for raising concern about national interests	L	S3	S1	S2
Facilitation of information-sharing platform	L/S1	S2	L/S1	S3
Detection				
Development of/enhancing algorithmic criteria for early detection of disinformation	S2	L	S1	S3
Fact/source-checking	S2	S3	L	S1
Response				
Strategic silence	L	S1	S2	S3
Strategic communication	L	S3	S1	S2
Sanctions and other economic and diplomatic measures	L	S3	S1	S2
Cyber information operations	L	S1	S3	S2
Flagging and dispelling	S3	L	S1	S2

### 4.2 Prevent, detect, and respond to AI-powered disinformation

From Ukraine’s perspective, the Law on Countering Disinformation could be justified as an emergency measure against internationally recognized Russian military aggression combined with hybrid warfare. However, this law should nonetheless be in line with international law and recognized principles of freedom of speech. We propose the classification of Ukrainian authorities’ powers with a set of preventative, detective, and responsive activities to address AI-enabled disinformation.

#### 4.2.1 Preventative actions

The development of a reliable news network is one of the first steps to take to prevent AI-powered disinformation. Our opinion is that democratic states should play a less active role in this activity than civil society organizations/traditional media, citizens, and VLOPs because of freedom of speech constitutional guarantees. Exceptions could be made only on the precondition of martial law limits. Strategies for raising awareness about AI-powered disinformation threats to national security should be among the measures for the state to undertake, in order to strengthen society’s compliance. As part of regulating and countering AI-powered disinformation, states should provide mechanisms to raise national interest concerns based upon academic research, while taking into consideration intelligence community analysis.

For Ukraine, such a procedure could involve CCD’s proposals to NSDC based on Security and Defense agencies’ analyses and civil society organizations/traditional media/academia inputs. The vital point of this mechanism is the implementation of NSDC decisions by VLOPs. Public-private cooperation in counter-disinformation requires knowledge-sharing between governments, VLOPs, and other stakeholders. An experience-based, lessons-learned platform, to share knowledge of adversaries’ methods and techniques, etc. can be developed in Ukraine, based on the example of disinfocloud.com, an online platform provided by the US Global Engagement Center to connect with relevant stakeholders (Global Engagement Center, 2024).

In the EU there is a different approach: an independent, non-profit organization focused on tackling sophisticated disinformation campaigns targeting the EU, its member states, and core institutions – Disinfo Lab (EU DisinfoLab, 2024). An important action in preventive measures of AI-powered disinformation is the ongoing education and awareness-raising agenda among the actors involved in combating disinformation. This includes a set of measures aimed at raising the level of media literacy and information hygiene among the population.

In an environment where the information space is filled with a large amount of destructive content, the ability to critically evaluate information becomes vital. For example, teaching citizens to distinguish facts from opinions or propaganda helps protect them from disinformation, including from that generated by AI. An important aspect of media literacy is also understanding the algorithmic mechanisms that govern the presentation of content on SM and news platforms, which allows for a better understanding of why people see certain content. Educational activities to improve media literacy and information hygiene should be systematic and cover all age groups. This can be done through educational programs at schools and universities, training for adults, as well as through the media and social networks. Particular attention should be paid to the younger generation, who are active users of digital technologies and are particularly vulnerable to disinformation. Successful implementation of these measures will contribute to the creation of a more resilient society that can effectively resist destructive information influences.

The Russian war against Ukraine has shown that media literacy is not only an academic topic for discussion but also an important process of developing relevant skills that save health and life. In general, the promotion of media literacy in Ukraine is part of a broader strategy aimed at creating an informed society. Recognizing these threats, Ukrainian authority has been actively engaged in public awareness programs and campaigns and cooperation with civil society organizations to promote media literacy as a tool to strengthen the country’s information resilience (Horban and Oliinyk, 2024).

Finally, scientists advocate for a global consensus on the ethical usage of GenAI and implementing cyber-wellness educational programs to enhance public awareness and resilience against disinformation (Shoaib et al., 2023).

#### 4.2.2 Detection

The best counter AI-powered disinformation response in SM, arguably, are algorithmic approaches to detecting disinformation before it becomes shareable. These actions require clear legal regulation of VLOPs responsibility to detect AI-powered disinformation. AI-powered disinformation campaigns can rarely be detected at the early stages, for instance, adversaries’ research on the audience or narratives and fake news preparation including making the AI-powered disinformation credible. Such activities can only be detected by clandestine operations of the intelligence communities.

However, VLOPs actually have the technical capabilities “*to detect mis- and disinformation in real time*” (Bharat, 2017). The basic criteria to qualify some activity as AI-powered disinformation could be if the activity: developed or disseminated by AI system; contains deceptive elements; has the intention to harm; is disruptive; constitutes interference” (Pamment et al., 2024). Such criteria must be available to the public, if used by detection tools.

In terms of impact, the detection of AI-powered disinformation could be made using AI, before fake news dissemination occurs in SM. VLOPs already use their AI-based products to provide feedback to commenters about potential perceived toxicity of content in real-time (for instance Jigsaw’s Perspective and Tune). This is a valuable tool for individuals, which allows readers to choose the level of toxicity they will see in comments across the internet (Jigsaw, 2024). Scientists like Smith et al. (2021) propose an end-to-end system to perform narrative detection, hostile influence operations account classification, network discovery, and estimation of hostile influence operations causal impact; as well as a method for detection and quantification of causal influence on a social network. Such results could be used by the Ukrainian authority to detect AI-powered disinformation.

The technical approach proposed by Nitzberg and Zysman (2022) for enabling AI to slow down the amplification of disinformation messages by the employment of time-limitation features for sharing suspected messages, could be efficient at a post-detection stage. If the message is not confirmed to contain fake elements, it could be disseminated at the usual pace; otherwise, it should be flagged or dispelled.

The next action to counter AI-powered disinformation is fact-checking. The authors propose that a fact-checking mechanism be used as a detection activity – before dissemination of what is suspected by AI to be false news. At present, fact-checking occurs after the incriminated fake news has been disseminated. This approach, however, is not sufficient. False information is diffused and has a “*continued influence effect*” (Lewandowsky et al., 2012). Thus, it is vital to develop a proactive counter-AI-powered disinformation detection mechanism. The fact-checking tools developed by VLOPs (mentioned above) and Ukrainian projects like StopFake (2024) and VoxUkraine (2024) etc., could all contribute to the Poynter Institute’s international network (IFCN Code of Principles, 2024).

There were and still are certain factors that could influence early detection of fake news: for instance, a debate about using encryption, particularly in VLOPs (National Academies of Sciences, Engineering, and Medicine, 2018). These debates seriously challenge counter AI-powered disinformation measures at the detection stage, from both a legal and technical perspective.

It may also be necessary for developers to employ identifiers that permit the identification of content produced by AI. One of the ways to counter the above-mentioned is to create special AI-based software that will mark the content in which the visual or audio part has been interfered. This will alert the user about the possible danger of using the “real-time” deepfake. However, the development of such software requires a long time and significant resources, which makes it impossible to counter this destructive influence. Therefore, the Ukrainian experience proposes to counteract “real-time” deepfake calls by carefully verifying the facts of the planned online meeting, staying in contact only through official means of communication, using different communication channels when organizing a video call, and following basic rules of cyber hygiene.

#### 4.2.3 Response actions

Even though attribution in AI-powered disinformation efforts might be challenging, it’s crucial to coordinate attribution and response when sufficient evidence is available and to publicly denounce those who spread false information (Kertysova, 2018). The response actions against AI-powered disinformation are dependent on the attribution of hostile influence campaigns, which is difficult. Response actions are also contingent on jurisdiction, which defines the mechanisms for decision-making as well as the status of data in transit. VLOPs, for instance, can change the data transactions from one jurisdiction to another using their servers’ locations and business process requirements. The same could be done by perpetrators to hide the tracks of disinformation dissemination. In consequence, this would severely complicate the attribution of AI-powered disinformation.

Ukrainian experience shows that one of the most important tools for responding to destructive information influence is the development of positive strategic narratives that help build society’s resilience to disinformation, particularly that powered by AI. These narratives strengthen trust in official sources and create a positive image of the state in the international arena. The development of such narratives involves the dissemination of new and reliable materials with the involvement of experts from academia, civil society, foreign language experts, media representatives, and partners from other democratic countries. This ensures a high level of diversity and reliability of information. In general, positive strategic narratives should be based on real achievements and events that build trust in information sources. They should be understandable and relatable to the audience, taking into account the values and interests of the latter. This case shows that positive strategic narratives are a powerful tool for countering AI-powered disinformation and strengthening information security and society’s resilience to external influences.

The same applies to the identification of negative (hostile) strategic narratives, which is crucial for countering AI-powered disinformation. Identifying dangerous messages aimed at discrediting state institutions, undermining trust in official sources, and creating panic among the population is a top priority. Analyzing the purpose and context of hostile narratives allows us to understand the goals behind the messages. Identifying the tactics and methods used in hostile narratives, such as intimidation, divergence, and fake news enables to develop of strategic countermeasures to neutralize their impact. It is worth noting that developing positive strategic narratives and identifying negative strategic narratives are the primary countermeasures against the influence of destructive information, as through such actions it becomes possible to identify global directions for countering AI-powered disinformation and the main steps toward it.

Another way to respond to AI-powered disinformation – disregard or strategic silence – should be considered in government decision-making taking into account that public opinion would have a tendency to forget quickly. Strategic silence could be used when the risk of danger for national security narratives to be perceived and believed by the population is low. However, this method carries significant potential risks. The main danger lies in the possible incorrect determination of the threat level. If the AI-powered disinformation that is decided to be ignored has a high level of disruptive impact, ignoring it can have serious consequences. For example, it may increase the spread of harmful narratives that can negatively affect public opinion, increase distrust of state institutions, or even cause panic. Therefore, the decision to use strategic silence should be made based on a thorough analysis of the potential impact of AI-powered disinformation. It is important to take into account not only the current state of public opinion but also the potential long-term consequences that may arise from underestimating the threat.

Strategic communication as a way to respond to AI-powered disinformation aims to provide and disseminate new and truthful content; this approach requires time, resources and a systemic framework. The use of humor as a part of responding to disinformation will also help to increase the dissemination of counter disinformation messages on SM platforms. Sanctions and other economic and diplomatic measures are additional legal tools to respond to AI-powered disinformation. One example of a sanction is the US legislation mandating the sale of TikTok based on concerns over disinformation and foreign propaganda (Fung, 2024).

Informational sanctions (flagging or blocking SM accounts) is an approach proposed by the authors, for further consideration and possible use against entities and individuals involved in AI-powered disinformation. In the context of the implementation of the information sanctions mechanism in Ukraine, it is necessary to emphasize a number of important tasks of the CCD at the NDC, including analysis and monitoring of events and phenomena in the country’s information space, assessment of the state of information security and analysis of Ukraine’s presence in the global information space. One of the key aspects of the CCD’s activities is the identification and study of current and predicted threats to Ukraine’s information security.

Rapid identification of the main actors generating AI-powered disinformation is crucial for an effective countering of information threats. The CCD closely cooperates with state authorities, law enforcement, and intelligence agencies, including foreign ones, to provide selected and analyzed data on key actors generating AI-powered disinformation. This data is passed on to the appropriate authorities for imposing sanctions and decision-making.

This innovative model of work of the CCD provides a comprehensive approach to countering AI-powered disinformation. It includes not only the identification and analysis of threats but also active cooperation with various national and international organizations, which allows them to respond quickly to changes in the information environment and effectively counter AI-powered disinformation campaigns. In particular, the CCD uses modern technologies to monitor the information space, analyze large amounts of data, and predict potential threats. This includes the use of AI algorithms to automatically detect anomalies in media content. The results of such analysis allow us to accurately identify sources of AI-powered disinformation and assess their impact on society.

Response in the form of cyber information operations is conducted covertly. The malicious use of general-purpose AI for deception and public opinion manipulation is a further topic of concern. AI-powered disinformation can be produced by adversaries and spread more easily even with the aim of influencing political processes.

Response actions include flagging and dispelling fake messages in SM. However, flagging or labeling fake information as “disputed” is not successful because it causes more sharing of the flagged content, and merely labeling information as fake does not lead to a reduction in its spread (Smith, 2017). One of the proposed ways to address the issue of deepfakes is to create a digital watermarking system that can verify the authenticity of media content (Thumos, 2024). Watermarking, which employs an invisible signature to identify digital content as coming from or being updated by AI, is one recommended technique for spotting disinformation (Christ et al., 2024). Although they are helpful, technical countermeasures like content watermarking are typically vulnerable to reasonably skilled offenders (AI Safety Institute, 2024).

The extent of governmental authority to counteract AI-powered disinformation with respect to freedom of speech, privacy, and rule of law principles is shown in Table 1.

Authors assign each action mentioned in the table to stakeholders based upon the following considerations: (1) the state cannot exercise influence on the development of a reliable news network and the fact/source-checking process, apart from the official governmental platform; (2) developing and enhancing algorithmic criteria for early detection of AI-powered disinformation, as well as flagging and dispelling it, are the responsibility of VLOPs due to their technical capacity; (3) the state authority should be able to choose the proper response to AI-powered disinformation in order to counter it (excluding flagging and dispelling).

## 5 Conclusion

AI-powered disinformation is becoming increasingly present in our lives and addressing it should be high on the agenda of national governments and interstate entities. Specifically, the legal means must be adjusted, based on detailed analyses of counter disinformation ecosystem, international and national legislation, as well as emerging regulations on AI systems. The European Media Freedom Act, the Future of EU Digital Policy, the EU Code of Practice on Disinformation, and the European AI Act already contain some norms for the regulation of AI-powered disinformation. When it comes to the work of very large online platforms (VLOPs), their internal counter-disinformation policies are largely oriented on the US liberal legislation in counter-disinformation, as most VLOPs are headquartered in the U.S.

Amid these realities, the transformations taking place in Ukraine present a case of particular interest. The country’s government is actively harmonizing its legislation with the EU binding legislation and regulations in AI- and counter-disinformation measures. Ukrainian Law on counter-disinformation measures, developed as an emergency response to internationally recognized Russian military aggression and hybrid warfare tactics, underscores the crucial need to align even emergency measures with international law and principles of free speech.

The authors proposed a set of preventative actions. These are developing reliable news networks, raising awareness, providing a mechanism for raising concerns about national interests, and facilitating information-sharing platforms. Detection actions are defined as developing/enhancing algorithmic criteria for early detection of disinformation, and fact/source-checking. Response actions are defined as strategic silence, strategic communication, sanctions and other economic and diplomatic measures, cyber information operations, and flagging and dispelling.

## Footnotes

^1^p. 110.

^2^p. 60 of the AI Act.

^3^p. 120.

^4^p. 134.
